# Male Green Mud Crab (*Scylla paramamosain*) Is More Vulnerable to Handling Stress than Female: A Comprehensive Insight into Physiological Changes and Health Status

**DOI:** 10.3390/ani16193143

**Published:** 2026-10-08

**Authors:** Noah Esmaeili, Chenyang Wu, Cong Yin, Peixun Chen, Jinsheng Zhou, Hongyu Ma

**Affiliations:** 1 Guangdong Provincial Key Laboratory of Marine Biotechnology, Shantou University, Shantou 515063, China; noah.esmaeili@utas.edu.au (N.E.); wcyang911@163.com (C.W.); 24cyin@stu.edu.cn (C.Y.); chenpeixun2026@163.com (P.C.); 20jszhou@stu.edu.cn (J.Z.); 2 International Joint Research Center for the Development and Utilization of Important Mariculture Varieties Surrounding the South China Sea Region, Shantou University, Shantou 515063, China; 3 STU-UMT Joint Shellfish Research Laboratory, Shantou University, Shantou 515063, China

**Keywords:** aggressive behavior, eustress, mild stress, stress adaptation, stress programming

## Abstract

The current research investigated the effects of repeated mild handling stress (RMH) and acute handling stress (AHS) in male and female green mud crabs *(Scylla paramamosain*) on production, hemolymph biochemistry, immune system, antioxidant defense, endoplasmic reticulum stress gene expression, stress response, energy, and ion homeostasis. Gill, hepatopancreas, hemolymph, and muscle of 4 groups (F, M, F-RMH, and M-RMH) were sampled after 60 days of farming and after 27 h of AHS. No significant difference in growth performance and survival rate after 60 days was identified among the groups. Habituation in the M-RMH groups was observed (as shown by behavior change) and resulted in reduced fighting and broken legs. The lowest survival rate (9.09%) after AHS was observed in M crabs, which coincided with lower values of total protein in the gill, lysozyme, peroxidase, total antioxidant capacity, and glucose in the hemolymph, and higher levels of total amino acid, lactate dehydrogenase, sodium, and cholesterol in the hemolymph. Habituation may mitigate stress and aggression, which are significant issues in crab aquaculture.

## Simple Summary

Mild stress at the right time and in the right manner can lead to habituation and eustress. The present study for the first time tested the effect of repeated mild handling and acute handling stress (AHS: sampling 3 h after the last stress event) on the production, hemolymph biochemistry, immune system, antioxidant defense, stress response, energy and ion homeostasis, and gene expression related to endoplasmic reticulum stress in male and female green mud crabs. We sampled the gill, hepatopancreas, and muscle of four groups after 60 days of the experiment and after 27 h of AHS stress for the above-mentioned parameters. Nonetheless, males exhibited a decreased survival rate compared to females following 8 days of AHS stress. The RMH groups habituated, showing reduced fighting and broken legs in males compared to controls, but not in terms of survival rate. The lowest survival rate after AHS was related to the male control (M) crabs, which coincided with lower values of total protein in the gill, lysozyme, peroxidase, total antioxidant capacity in the hemolymph, and glucose, and higher levels of lactate dehydrogenase, sodium, and cholesterol.

## 1. Introduction

Rising global seafood consumption, customer demand for seafood delicacies, and the ongoing diversification in aquatic species products are factors bolstering the growth of the global crab market. Crab aquaculture has been boosted in recent decades and has reached 1.2 million tons [1]. The green mud crab (*Scylla paramamosain*) is the predominant aquacultured crab species due to its rapid development, substantial size, high meat yield, palatability, elevated local and worldwide market values, and adaptability to various farming systems [2]. However, there are several obstacles to the sustainable development of crab aquaculture. Among them, husbandry stressors, such as handling stress, are significant stressors for several crab species.

Handling stress is one of the most prevalent and inevitable stressors in aquaculture practice and experimental research. This stressor can be either acute and short-term, causing a transient disruption in homeostasis, or chronic, affecting the longer-term growth, physiology, health, and reproduction [3,4]. Research on crustaceans revealed that handling green shore crabs (*Carcinus maenas*) for 10–15 min every 2 weeks for 2 months increased mortality by up to 60%, while the unhandled group’s mortality was just 3% [5]. Additional investigations on crabs have demonstrated how handling stress affects growth, welfare, hemolymph biochemical balance, behavior, immunity, and the antioxidant system [3,4,6,7]. However, handling stress may not be harmful if it can be done quickly and gently. After 40 days, Chinese shrimp (*Fenneropenaeus chinensis*) raised at high stocking densities showed enhanced survival and growth under mild net-chasing stress (trained 3 times a day under 25% of exhaustion) without compromising their health or behavior [8]. Through behavioral modulation, net-chasing stress (less than 25% of exhaustion) also enhanced the size homogeneity of freshwater prawns (*Macrobrachium rosenbergii*) raised at high stocking density [9].

Stress at the right time and manner can induce habituation. Habituation refers to the capacity of animals to exhibit adaptive responses to stressors, resulting in reduced reactivity and sensitivity to recurring stressors, which cannot be explained by sensory adaptation or motor fatigue [10]. These principles are discussed in the concept of behavior, and in terms of aquaculture, habituation can reduce stress response and improve survival rate. If habituation successfully occurs through repeated mild stress, eustress happens. Mild stress may help an animal cope with subsequent stressful situations and prepare it for future stressful conditions. Habituation with various stressors, such as stocking density, chasing stress, and hypoxia in fish, was observed [11,12,13,14]. However, other researchers with the same above-mentioned stressors did not observe the occurrence of habituation [15,16,17]. Studies in fish and crustaceans showed that eustress and distress (negative stress) elicit various responses in the body regarding the neuroendocrine system, behavior, and other physiological mechanisms [18,19,20,21,22,23,24]. This shows that the experimental design, species, kind of stressors, severity, and other parameters affect the occurrence of habituation and eustress.

To the best of our knowledge, no studies have been conducted on how long-term mild handling stress affects physiological processes. The only available study is from our investigation, which, through transcriptomics, found that energy production, tyrosine metabolism, amino acid metabolism, and deoxyribonucleic acid (DNA) replication were the most altered (mainly enriched) pathways under acute handling stress, while recurring moderate handling stress primarily affected gills. The male and female responded differently, and applied mild handling stress in the current project reduced the aggressiveness of the green mud crab [25]. The current investigation tested how repeated mild handling (RMH: net chasing and handling for 45 s (20% of exhaustion) every 4 days) can increase green mud crabs’ tolerance to acute handling (AHS: acute 4 min net chasing and handling stress every 12 h). We hypothesized that repeated mild handling training may positively enhance the green mud crab’s resilience to AHS. Accordingly, the immune system, antioxidant defense, hemolymph biochemistry, osmoregulation, energy homeostasis, stress response, and genes related to endoplasmic stress were measured in male and female green mud crabs.

## 2. Materials and Methods

### 2.1. Animal Ethics

All animal handling and experimental methods were conducted in strict adherence to ethical principles, as checked by the Animal Care and Use Committee of Shantou University and in accordance with the Guide for the Care and Use of Laboratory Animals.

### 2.2. Crab Farming

The trial was carried out in the aquaculture lab of Shantou University, using a vertical crab farm also referred to as a crab house. After 2 weeks of acclimation, crabs were farmed for 60 days. The details of farming conditions and crabs were reported earlier [25]. Accordingly, 112 healthy crabs (weight 29.5 ± 4.4 g), comprising 56 females and 56 males, were reared individually.

### 2.3. Experimental Treatments and Mild and Acute Programmed Stress

The four treatments consisted of female Control (F), male Control (M), female RMH (F-RMH), and male RMH (M-RMH). In RMH groups, crabs underwent net chasing and handling for 45 s (20% exhaustion) every 4 days, totaling 14 stress events throughout the 60-day trial duration. We initially measured how long it took crabs to become fatigued from chasing nets and managing stress, and it was around 230 s. The crab was considered exhausted when it stopped trying to escape and did not react to prodding for 10 s. Feed intake was not discontinued the following day, with the brief chasing stress (20% of exhaustion), which was an indicator that the stress had been mild. The crab fasted for a full day before sampling [25]. Every crab was weighed and measured for carapace length, width, and growth performance [25]. Three samples (3 crabs) were obtained from each tissue (muscle, gills, and hepatopancreas). In order to provide adequate hemolymph for laboratory examination, 3 additional samples were taken from each group, and hemolymph from 2 crabs from each treatment were pooled to make 3 final representative samples.

Using a 2 mL syringe (without anticoagulant), the hemolymph of each crab was extracted from the base of its walking legs and refrigerated for 8 to 10 h. After crushing the hemolymph clot with sterile surgical forceps, the mixture was centrifuged at 10,000× *g* for 15 min at 4 °C. Then, the hemolymph supernatant was immediately collected and stored at −80 °C. The remaining 15 crabs from each treatment were then moved to tanks for acute handling and chasing (AHS: 4 min net chasing and handling stress every 12 h, 3 stress events). The 60 crabs were subjected to AHS, and we placed three crabs in each tank, with at least one crab of each sex that was around the same size. A net was used to chase the crabs in the aquarium and, if feasible, catch them two or three times. For each of the four groups, we ran the chasing stress with the same net and speed. We sampled the hepatopancreas, gills, muscle (3 crabs from each group), and hemolymph (3 more crabs from each treatment) for additional analysis 3 h after the last stress event. Samples from every group were collected roughly 27 h after beginning the AHS. For 8 days, the AHS was applied every 12 h to the remaining 36 crabs. During these 8 days, the crabs were not fed to avoid interference. Each tank included 3 crabs, ensuring at least one of each sex. Upon the death of a crab, it was replaced with another distressed crab from the same treatment to maintain consistent density and social contact. Replacement animals were included in the survival calculations. Testing the crabs’ ability to survive the AHS after 8 days was the goal. The hemolymph pooling was conducted similarly to the end of the experiment (Supplementary file (Figure S1)). For experimental analysis, all of the samples were placed in RNAkeeper, frozen in liquid nitrogen, and kept at −80 °C. To ensure uniformity, the groups were compared using tissues from the same crabs [25].

### 2.4. Antioxidant Defense and Immune System Parameters

The hepatopancreas, gill, and muscle tissue from each crab were weighed (500 mg from each individual tissue) and then homogenized with ice-cold phosphate buffer in a tissue: buffer ratio of 1:7 using an IKA homogenizer (T10B, IKA Company, Staufen, Germany). The resulting homogenate was centrifuged at 10,000× *g*, 4 °C for 30 min, and the supernatant was immediately collected for the determination of the parameters.

The resultant supernatant was examined right away to measure the Na^+^/K^+^-ATPase activity, total protein, glycogen, adenosine triphosphate (ATP), lysozyme, acid phosphatase (ACP), total nitric oxide synthase (NOS), protein carbonyl, total superoxide dismutase (SOD), catalase, malondialdehyde (MDA), glutathione peroxidases (GPx), glutathione transferases (GST), total antioxidant capacity (T-AOC), and alkaline phosphatase (ALP) using available protocols in the commercial kits (Beijing Solarbio Science and Technology (Beijing, China) and Nanjing Jiancheng Bioengineering Institute (Nanjing, China)). The supernatant of hemolymph was also used to measure ions (sodium, potassium, calcium, magnesium, chloride), aspartate aminotransferase (AST), alanine aminotransferase (ALT), lactate dehydrogenase (LDH), peroxidase, glucose, urea nitrogen, lactate, total ammonia, total amino acids, total protein, triglyceride, total cholesterol, and uric acid using the above-mentioned kits. Crustacean hyperglycemic hormone (CHH) was measured with an ELISA kit from Coibo Bio (Shanghai, China) based on the available protocol.

### 2.5. Gene Expression Analysis

The 9 genes related to endoplasmic reticulum (ER) stress, including C/EBP homologous protein (*CHOP*), activating transcription factor 6 (*ATF6*), inositol-requiring protein-1 (*IRE1*), eukaryotic translation initiation factor 2-alpha (*EIF2α*), X-box binding protein 1 (*XBP1*), protein kinase RNA-like ER kinase (*PERK*), endoplasmic reticulum oxidoreductase-1 (*ERO1α*), immunoglobulin heavy chain binding protein (*BIP*), and activating transcription factor 4 (*ATF4*) were investigated. Total ribonucleic acid (RNA) was extracted from 3 distinct tissues (gill, muscle, and hepatopancreas) of green mud crab, with each tissue sampled from a separate crab to ensure biological independence. Following dissection, tissues were placed in RNAkeeper (Vazyme, Nanjing, China) and kept at –80 °C until further processing. AG RNAex Pro RNA Extraction Reagent (Accurate Biology, Changsha, Hunan, China) was used for RNA isolation in accordance with the manufacturer’s instructions. The integrity of RNA was verified by electrophoresis on a 1% agarose gel. The concentration and purity of RNA were assessed utilizing a Thermo Scientific NanoDrop One spectrophotometer (Thermo Fisher Scientific, Waltham, MA, USA). RNA exhibiting acceptable purity (A260/A280 ratio between 1.8 and 2.2, and A260/A230 ratio > 2.0) was chosen for subsequent analysis and preserved at −80 °C. To synthesize cDNA, 1 μg of RNA and 5 μL HiScript IV All-in-one Ultra RT Super Mix for qPCR (Vazyme, Nanjing, China) and RNase-Free Water were added in a 20 μL reaction volume. Reverse transcription was conducted at 50 °C for 10 min, followed by 85 °C for 5 s to halt the reaction. Fifty percent of the generated cDNA was diluted 20-fold, and 1 μL of the diluted product was utilized for each qPCR assay.

The qPCR was conducted using a quantitative thermocycler, namely the Light Cycler 480 II Real-Time PCR System (Roche, Rotkreuz, Switzerland). The experiment adhered rigorously to the manufacturer’s guidelines specified in the ChamQ SYBR qPCR Master Mix kit (Vazyme Biotech, Nanjing, China). Each sample was subjected to three biological replicates and three technical replicates, with 18S rRNA as the reference gene for standardization. Each 10 μL reaction comprised 5 μL of 2× Master Mix, 0.2 μL of each primer (10 μM), 1 μL of diluted cDNA, and 3.6 μL of double-distilled water. The thermal cycling conditions for PCR were set as follows: pre-incubation at 95 °C for 30 s, followed by 40 cycles of 95 °C for 10 s and 60 °C for 15 s. Finally, the relative expression level of each gene was calculated using the 2^−ΔΔCt^ method [26]. Table 1 shows the genes, sequence (5′-3′), and their size (bp). The sources of the primers and information on their design were based on chromosome-level assembly and annotation (GenBank: JAYKKS000000000; RefSeq: GCF_035594125.1; BioProject: PRJNA1059155) and were reported elsewhere [27].

### 2.6. Statistical Analysis

After passing prerequisites such as normality, homogeneity of variances, and addressing outliers, the measured parameters were statistically analyzed with two-way ANOVA. The mean differences among samples for sex and RMH effect were considered. An independent-samples *t*-test was employed to compare the primary means derived from the two-way ANOVA comparing sex and RMH effects. Upon finding a significant interaction in the two-way ANOVA, we analyzed the original data, as illustrated in the figures. When the interaction was not significant, we then considered main effects (sex and RMH), which were reported in Table S1. The data before and after AHS were compared to discover the impact of acute stress, as was used earlier in a previous study [20]. All statistical analyses were performed with SPSS software version 27. *p*-values less than 0.05 were deemed statistically significant [20].

## 3. Results

### 3.1. Growth Performance and Behavioral Observation

The results of growth and behavioral observations were also reported in the previous article from this project [25]. In summary, RMH did not influence production parameters after 60 days; however, sex had a substantial impact on survival rate following 8 days of AHS. Accordingly, based on the pooled data extracted from two-way ANOVA, the survival rate for males (20.83%) was markedly inferior to that of females (54.17%). The survival rates of the F, F-RMH, M, and M-RMH groups after 8 days of AHS were 58.33%, 50.00%, 9.09%, and 33.33%, respectively. In the last 3 weeks of the experiment, the crab RMH group, especially the males, showed less reactive and fighting behavior when they were chased by the net inside the tanks. Additionally, under AHS, a reduced number of crabs in the RMH groups were attached to the door of the boxes compared to the Control group (5 versus 15). As the number of crabs was allocated to 3 per tank during AHS, they began to engage in aggressive behavior and subsequently discarded their chelipeds. The number of dropped chelae in the Control and RMH groups was 9 and 1, respectively [25].

### 3.2. Hemolymph Biochemistry and Total Protein in Tissues

**Figure 1:**
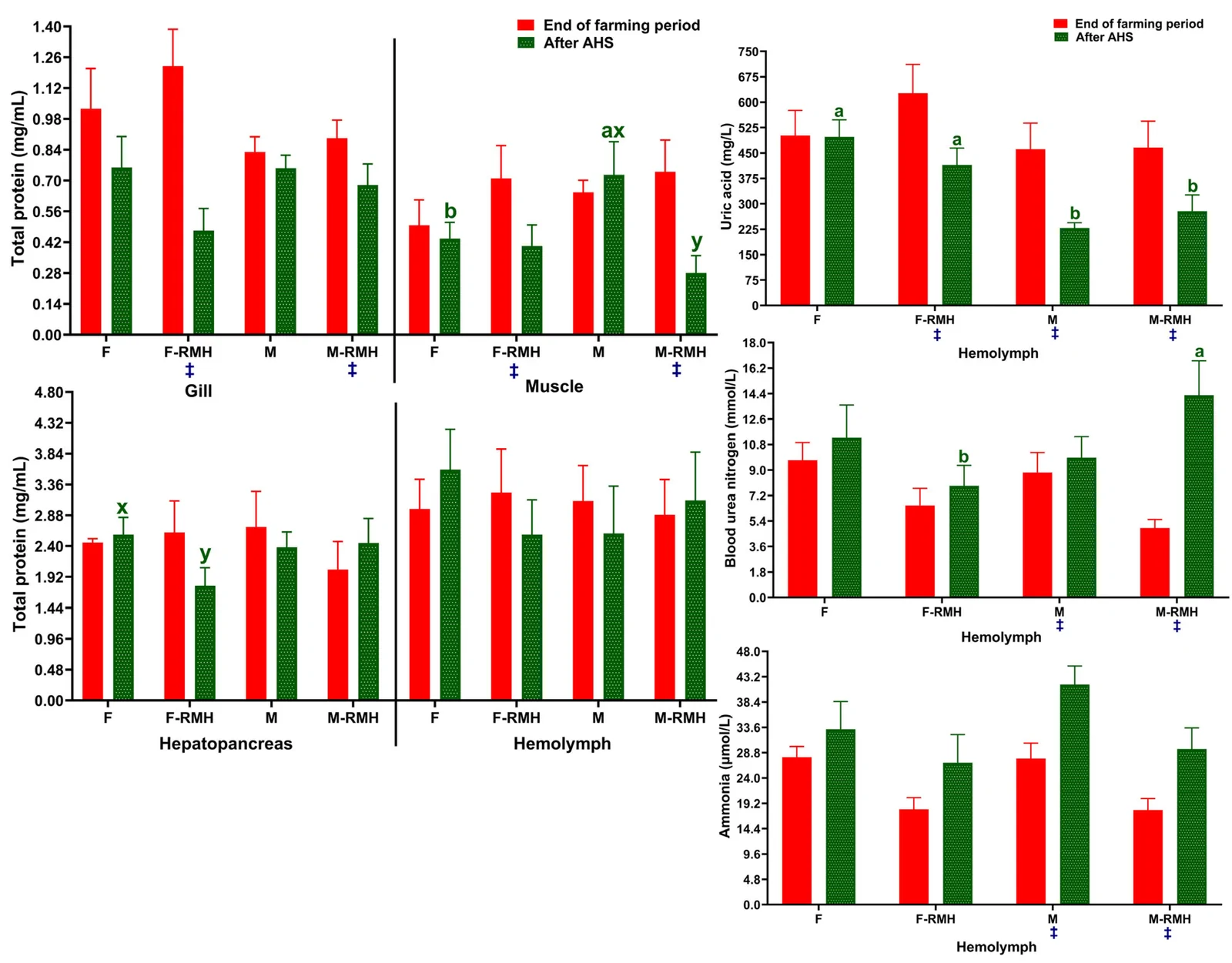
Hemolymph biochemistry results in green mud crabs reared under mild handling stress (RMH) and then exposed to acute handling stress (AHS). Values (mean ± SD) are from three samples per treatment, from three tanks. Each sample represents a tank, which was made by pooling two crab samples from each tank. Statistical analysis was performed at the level of the tank. Means without letter labels are not significantly different based on two-way ANOVA, and when the interaction was significant, the data were unpacked in the figures. The letters a and b indicate a significant difference between female (F) vs. male (M) or female-RMH (F-RMH) vs. male-RMH (M-RMH). The letters x and y indicate a significant difference between F vs. F-RMH or M vs. M-RMH. The symbol (‡) indicates significant differences between each group before and after AHS based on an independent-samples *t*-test (*p* < 0.05).

The data related to total protein, urea nitrogen, uric acid, and total ammonia were reported in Figure 1. The results of the present study indicated that males had a lower value of gill total protein than females, and ammonia and blood urea nitrogen in the RMH group were lower than in the Control (Table S1) (*p* < 0.05).

After AHS, the gill total protein and ammonia in the RMH group were lower than those in the Control (Table S1). The interaction effect for hepatopancreas total protein, muscle total protein, uric acid, and blood urea nitrogen was significant (Figure 1). Higher muscle total protein in M compared to M-RMH and the F group; higher blood urea nitrogen in M-RMH compared to F-RMH animals; and higher uric acid in F and F-RMH treatment than in males and M-RMH crabs, respectively, were observed (*p* < 0.05).

Also, to see the direct effect of AHS, we compared the data before and after the acute stress. The AHS increased uric acid and blood urea nitrogen and decreased ammonia levels in male groups. The AHS also reduced gill total protein in the M-RMH group compared to before stress.

### 3.3. Immune System Parameters

**Figure 2:**
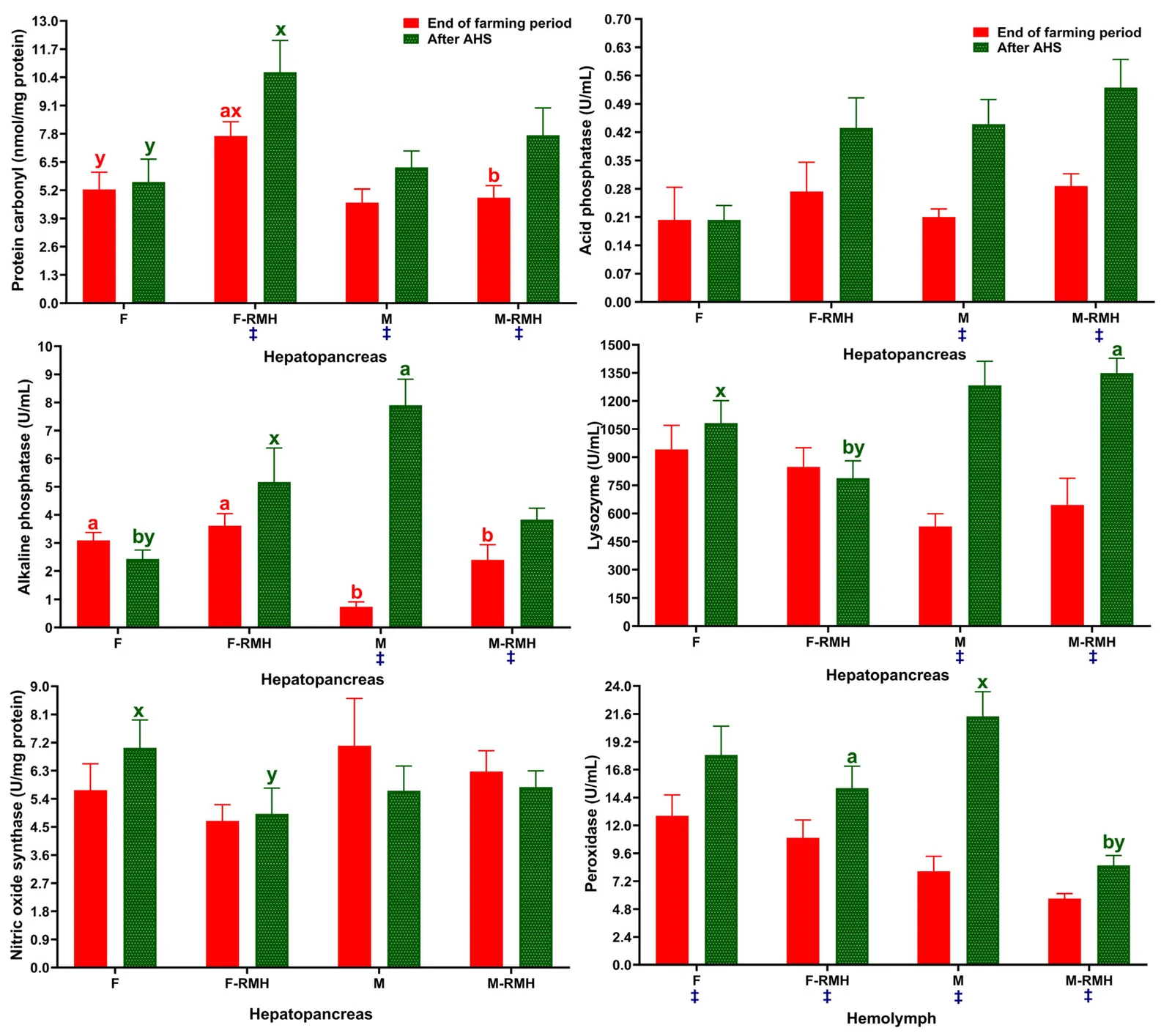
The results of immune system parameters in green mud crabs that were reared under mild handling stress (RMH) and then exposed to acute handling stress (AHS). Values (mean ± SD) are from 3 samples per treatment, from 3 tanks. Each sample represents 1 tank, which was made by pooling 2 crab samples from each tank. Statistical analysis was performed at the level of the tank. Means without letter labels are not significantly different based on two-way ANOVA, and when the interaction was significant, the data were unpacked in the figures. The letters a and b indicate a significant difference between female (F) vs. male (M) or female-RMH (F-RMH) vs. male-RMH (M-RMH). The letters x and y indicate a significant difference between F vs. F-RMH or M vs. M-RMH. The symbol (‡) indicates significant differences between each group before and after AHS based on an independent-samples *t*-test (*p* < 0.05).

The data related to the immune system, including NOS, peroxidase, lysozyme, protein carbonyl, ALP, and ACP, were reported in Figure 2. Table S1 indicates that males had lower values of lysozyme and peroxidase than females. Further, the NOS in males was lower than in females (*p* < 0.05). The interaction effect of two-way ANOVA for protein carbonyl and ALP was significant. The F-RMH group had higher levels of protein carbonyl than M-RMH and F animals, and this group also experienced an increase in ALP level compared to M-RMH crabs (*p* < 0.05).

After AHS, ACP values in the male and RMH groups were higher than in the female and Control treatments (*p* < 0.05). The interaction in the two-way ANOVA for lysozyme, NOS, peroxidase, protein carbonyl, and ALP was significant, and the data were unpacked in Figure 2. The F treatment exhibited a significantly lower value of ALP than the F-RMH and M groups. While lysozyme in the F-RMH group was reduced compared to the F and M-RMH crabs, the M-RMH treatment showed a lower value of peroxidase than the M and F-RMH groups (*p* < 0.05).

Also, to see the direct effect of AHS, we compared the data before and after this stressor. The results indicated that the M and M-RMH groups were affected more, so these groups had higher levels of protein carbonyl, ALP, ACP, lysozyme, and peroxidase after AHS than before this stress. Further, AHS elevated peroxidase in all crabs (*p* < 0.05).

### 3.4. Antioxidant Defense

**Figure 3:**
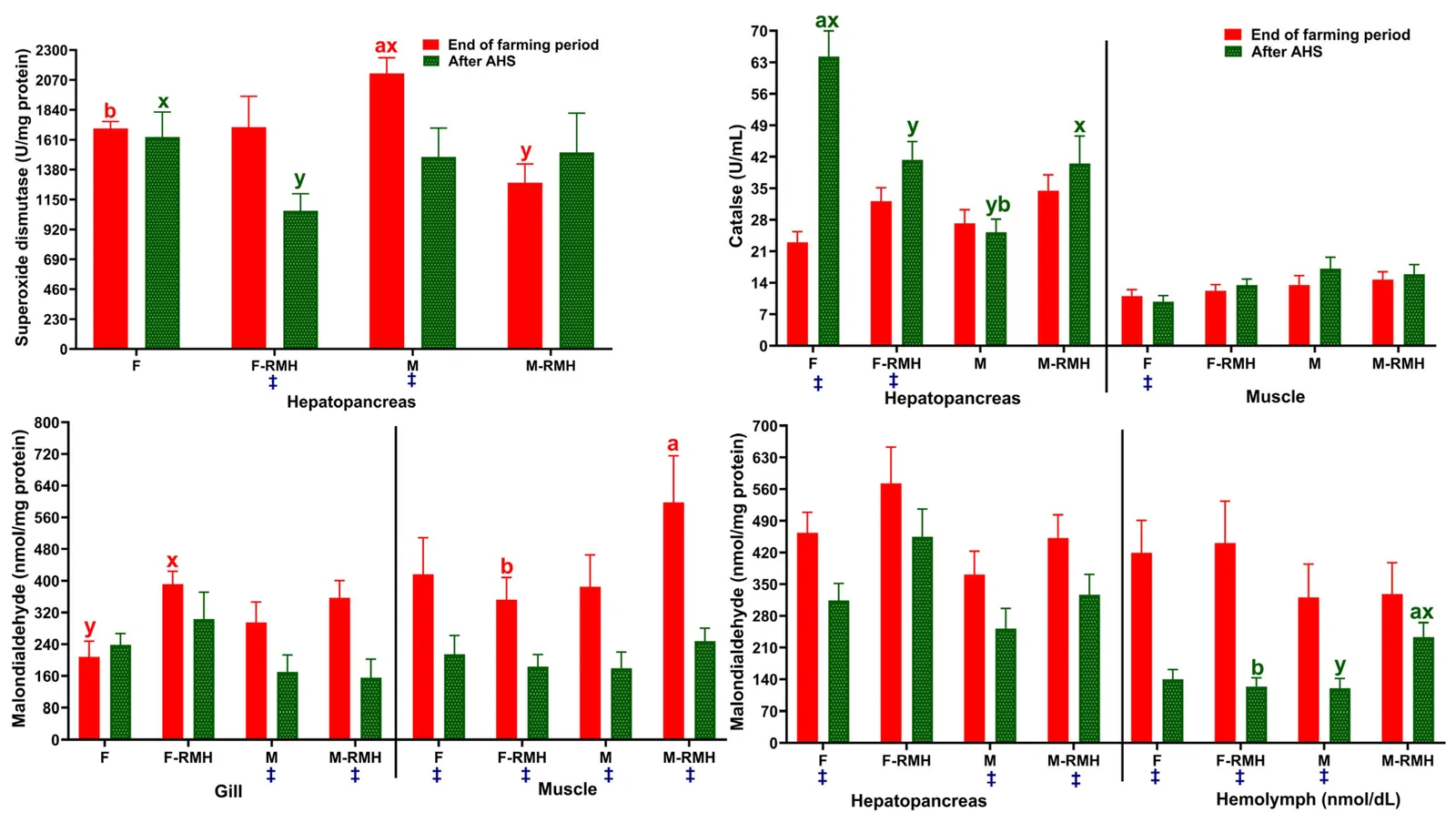
The results of antioxidant parameters in green mud crabs reared under mild handling stress (RMH) and then exposed to acute handling stress (AHS). The values (mean ± SD) are from three samples per treatment, from three tanks. Each sample represents a tank, which was made by pooling 2 crab samples from each tank. Statistical analysis was performed at the level of the tank. Means without letter labels are not significantly different based on two-way ANOVA, and when the interaction was significant, the data were unpacked in the figures. The letters a and b indicate a significant difference between female (F) vs. male (M) or female-RMH (F-RMH) vs. male-RMH (M-RMH). The letters x and y indicate a significant difference between F vs. F-RMH or M vs. M-RMH. The symbol (‡) indicates significant differences between each group before and after AHS based on an independent-samples *t*-test (*p* < 0.05).

**Figure 4:**
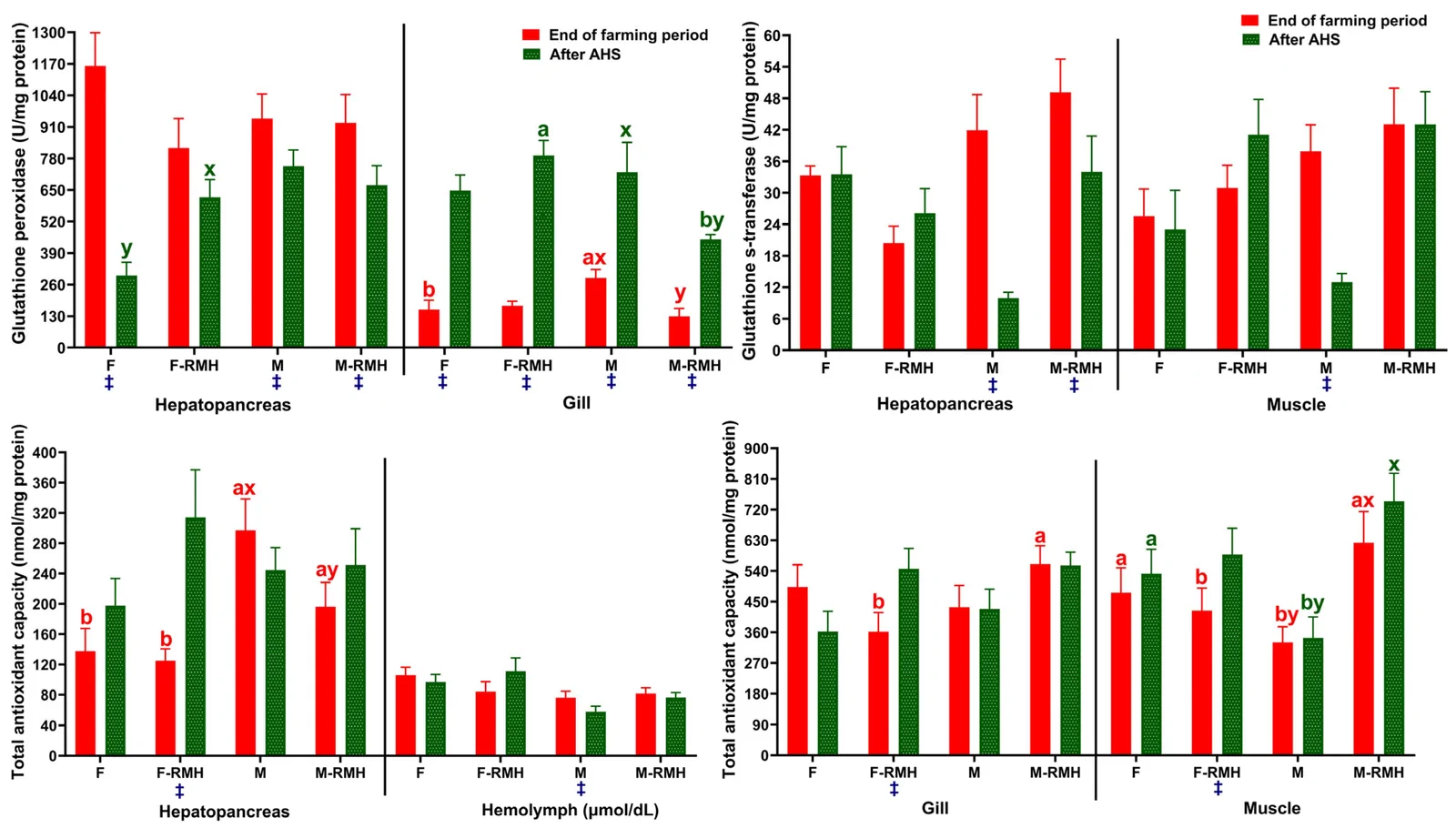
The results of antioxidant parameters in green mud crabs reared under mild handling stress (RMH) and then exposed to acute handling stress (AHS). Values (mean ± SD) are from 3 samples per treatment, from 3 tanks. Each sample represents 1 tank, which was made by pooling 2 crab samples from each tank. Statistical analysis was performed at the level of the tank. Means without letter labels are not significantly different based on two-way ANOVA, and when the interaction was significant, the data were unpacked in the figures. The letters a and b indicate a significant difference between female (F) vs. male (M) or female-RMH (F-RMH) vs. male-RMH (M-RMH). The letters x and y indicate a significant difference between F vs. F-RMH or M vs. M-RMH. The symbol (‡) indicates significant differences between each group before and after AHS based on an independent-samples *t*-test (*p* < 0.05).

Antioxidant system indicators, including SOD, catalase, MDA, GPx, GST, and T-AOC from this study, are reported in Figure 3 and Figure 4. Table S1 indicates that the RMH effect for hepatopancreas CAT, hepatopancreas MDA, and hepatopancreas GPx was significant. Regarding sex, males showed higher values of muscle CAT and muscle GST, and lower levels of hemolymph MDA, hepatopancreas MDA, and hemolymph T-AOC compared to females (*p* < 0.05). The interaction effect for SOD, hepatopancreas T-AOC, gill T-AOC, and muscle T-AOC was significant. The M group had significantly higher values of SOD, gill GPx, and hepatopancreas T-AOC than the M-RMH and F treatments. The M-RMH group also showed elevation of muscle MDA, hepatopancreas T-AOC, and muscle T-AOC in comparison with F-RMH animals (*p* < 0.05).

After AHS, while CAT level in muscle was elevated in males, gill MDA, hepatopancreas MDA, and hemolymph T-AOC in males were lower than in females (*p* < 0.05). Further, RMH groups showed increased levels of hepatopancreas MDA, muscle GST, and T-AOC in the hepatopancreas, hemolymph, and gill than the Control (*p* < 0.05). The interaction effects for several parameters were significant. The most important change was related to higher catalase in the F treatment than in the F-RMH and M groups, and a significantly higher level of muscle T-AOC in F crabs compared to the M group was observed. The M-RMH group also showed higher hemolymph MDA and lower GPx values in the gill than the M and F-RMH treatments (*p* < 0.05).

The AHS in the M group decreased SOD and MDA levels in the 4 tissues, GPx in the hepatopancreas, T-AOC in the hemolymph, and GST in the hepatopancreas and muscle (*p* < 0.05). Further, this stress significantly increased hepatopancreas catalase and gill GPx and decreased MDA levels in the hepatopancreas, hemolymph, and muscle in the F group.

### 3.5. Animal Response to Stress

**Figure 5:**
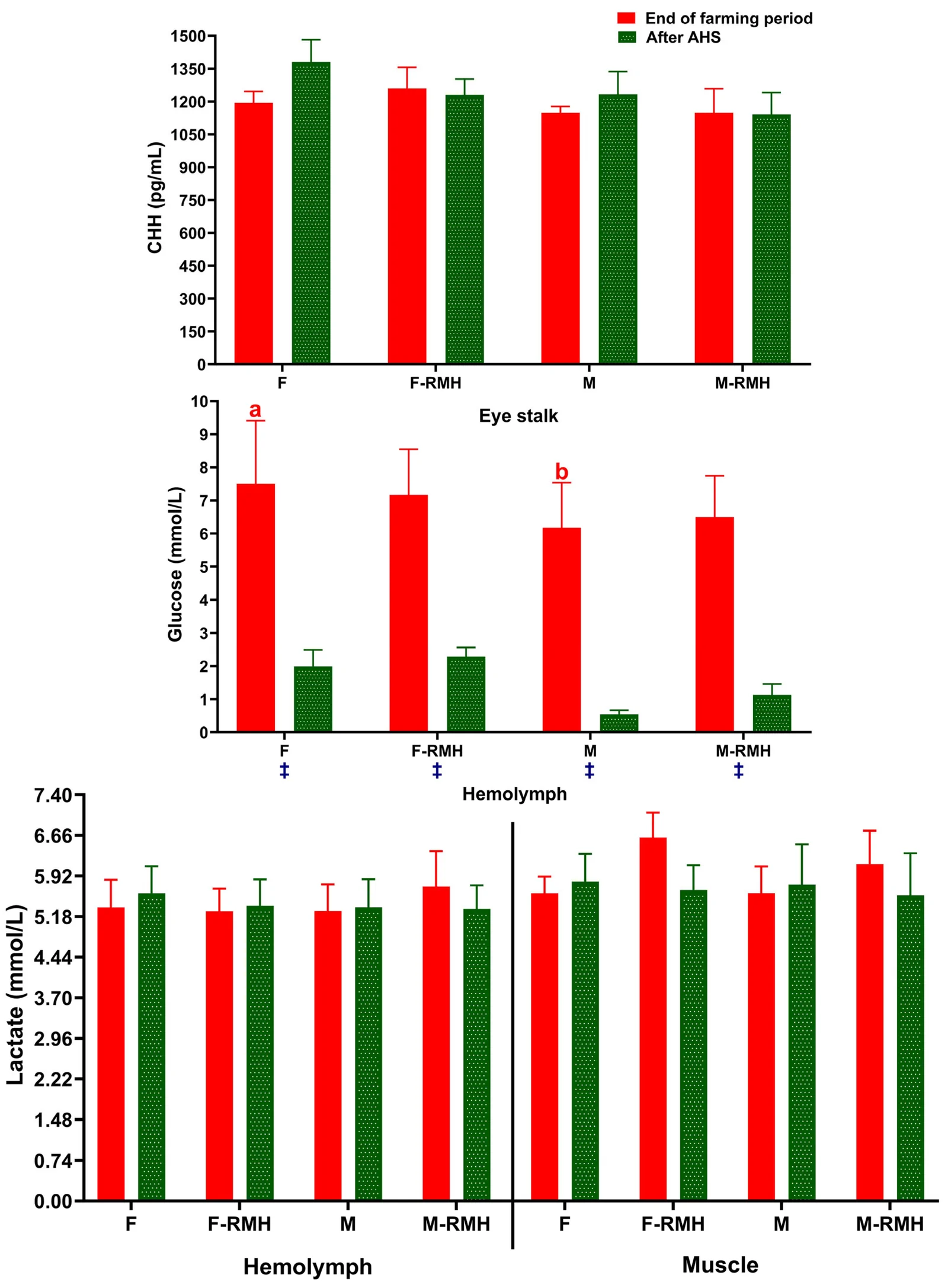
The results of stress parameters in green mud crabs reared under mild handling stress (RMH) and then exposed to acute handling stress (AHS). Values (mean ± SD) are from three samples per treatment, from three tanks. Each sample represents a tank, which was made by pooling two crab samples from each tank. Statistical analysis was performed at the level of the tank. Means without letter labels are not significantly different based on two-way ANOVA, and when the interaction was significant, the data were unpacked in the figures. The letters a and b indicate a significant difference between female (F) vs. male (M) or female-RMH (F-RMH) vs. male-RMH (M-RMH). The letters x and y indicate a significant difference between F vs. F-RMH or M vs. M-RMH. The symbol (‡) indicates significant differences between each group before and after AHS based on an independent-samples *t*-test (*p* < 0.05).

Stress parameters, including CHH, glucose, and lactate activity from the present research, are reported in Figure 5. The effect of RMH was significant for muscle lactate after 60 days of farming, and the RMH group had a significantly higher value of this parameter. The interaction effect for glucose was significant, so the F group had a higher value than the F-RMH and M crabs after the farming period (*p* < 0.05).

After AHS, males and Controls had lower values of glucose than females and the RMH groups, respectively (*p* < 0.05). The AHS also dramatically depleted the glucose level in all four groups (*p* < 0.05).

### 3.6. Homeostasis of Energy 

**Figure 6:**
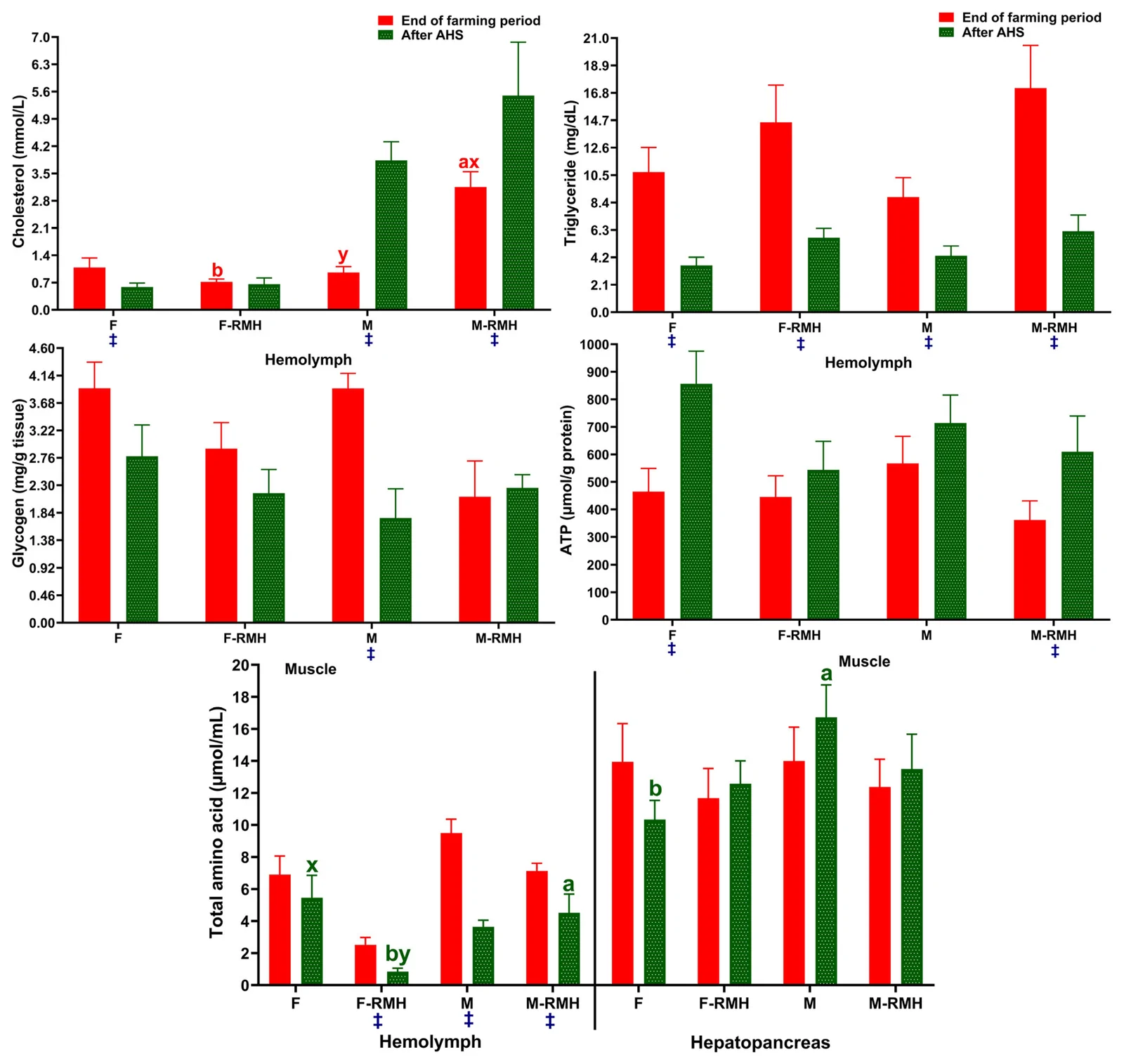
The results of energy homeostasis parameters in green mud crabs reared under mild handling stress (RMH) and then exposed to acute handling stress (AHS). Values (mean ± SD) are from three samples per treatment, from three tanks. Each sample represents a tank, which was made by pooling two crab samples from each tank. Statistical analysis was performed at the level of the tank. Means without letter labels are not significantly different based on two-way ANOVA, and when the interaction was significant, the data were unpacked in the figures. The letters a and b indicate a significant difference between female (F) vs. male (M) or female-RMH (F-RMH) vs. male-RMH (M-RMH). The letters x and y indicate a significant difference between F vs. F-RMH or M vs. M-RMH. The symbol (‡) indicates significant differences between each group before and after AHS based on an independent-samples *t*-test (*p* < 0.05).

The data related to cholesterol, triglyceride, glycogen, ATP, and total amino acids were reported in Figure 6. After 60 days, the hepatopancreas total amino acids in males were significantly higher than in females. ATP, total hemolymph amino acids, and glycogen were higher, and the triglyceride level was lower in the Control group compared to the RMH treatment. The interaction effect for cholesterol was significant, and the M-RMH group had a higher value compared to M and F-RMH treatments (*p* < 0.05; Table S1).

After AHS, males had a higher level of cholesterol than females. Triglyceride and ATP levels followed changes in the pre-stress period, and the Control group had lower and higher levels, respectively, when compared to the RMH group (*p* < 0.05). The interaction effect for hemolymph and hepatopancreas total amino acid was significant. Further, the F-RMH treatment had lower levels of hemolymph total amino acid than the F and M-RMH groups (*p* < 0.05). The AHS stressor increased cholesterol and decreased triglycerides in the M and M-RMH groups and also decreased glycogen levels in the M animals (*p* < 0.05).

### 3.7. Serological Enzymes

**Figure 7:**
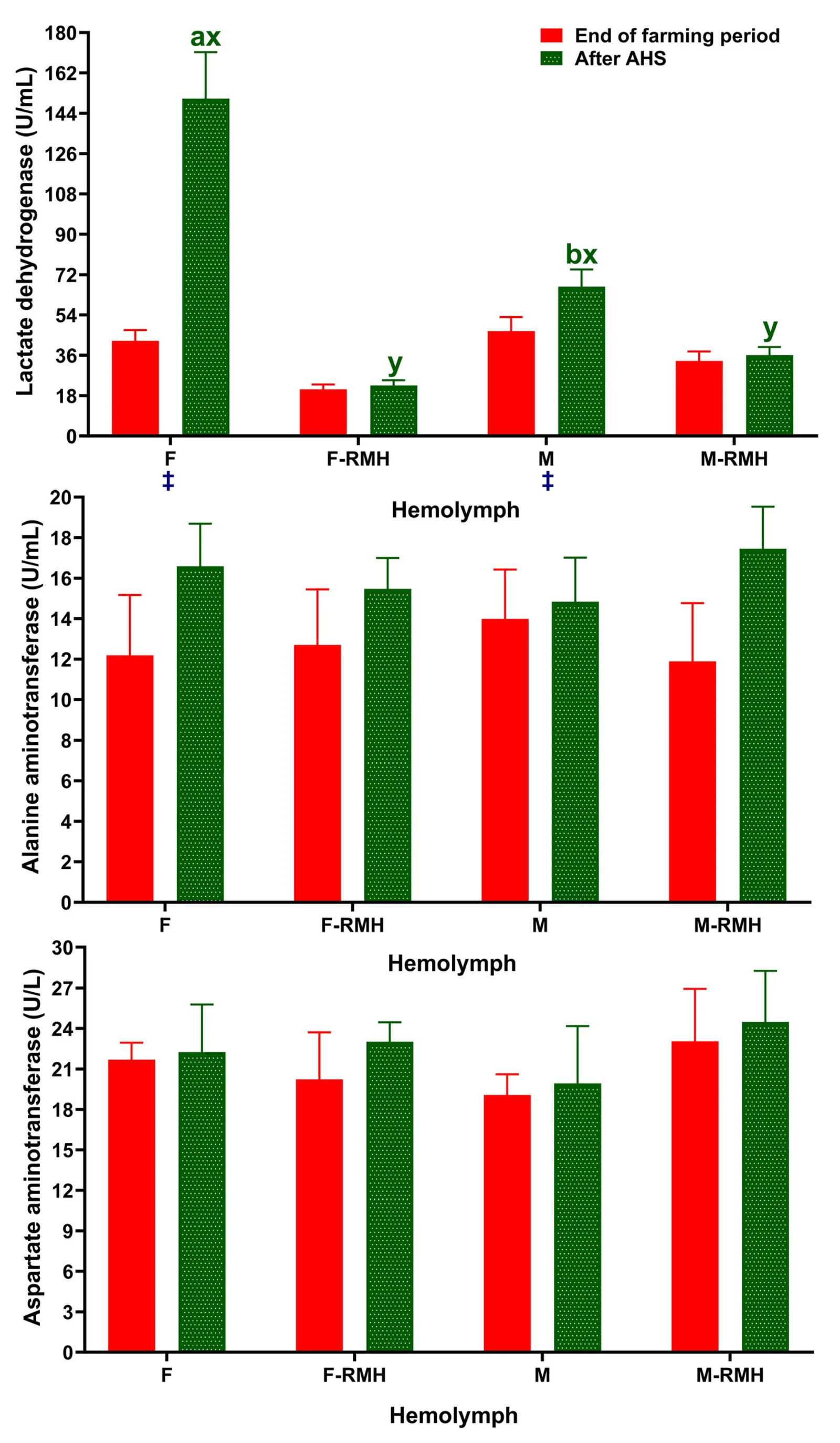
The results of serological enzyme parameters in green mud crabs reared under mild handling stress (RMH) and then exposed to acute handling stress (AHS). Values (mean ± SD) are from three samples per treatment, from three tanks. Each sample represents a tank, which was made by pooling two crab samples from each tank. Statistical analysis was performed at the level of the tank. Means without letter labels are not significantly different based on two-way ANOVA, and when the interaction was significant, the data were unpacked in the figures. The letters a and b indicate a significant difference between female (F) vs. male (M) or female-RMH (F-RMH) vs. male-RMH (M-RMH). The letters x and y indicate a significant difference between F vs. F-RMH or M vs. M-RMH. The symbol (‡) indicates significant differences between each group before and after AHS based on an independent-samples *t*-test (*p* < 0.05).

Figure 7 indicates data related to LDH, ALT, and AST in the hemolymph of the green mud crab. The effect of mild handling stress was significant for the LDH and RMH groups, and females had lower levels than the Control and male groups, respectively (*p* < 0.05). After AHS, the interaction effect for LDH was also significant, and the F group had significantly higher levels compared to F-RMH and M treatments. Also, M-RMH crabs experienced a decrease in LDH level compared to the M group, and higher levels of this parameter than the F-RMH group were observed (*p* < 0.05). Further, AHS significantly elevated this parameter in the F and M groups.

### 3.8. Homeostasis of Ions

**Figure 8:**
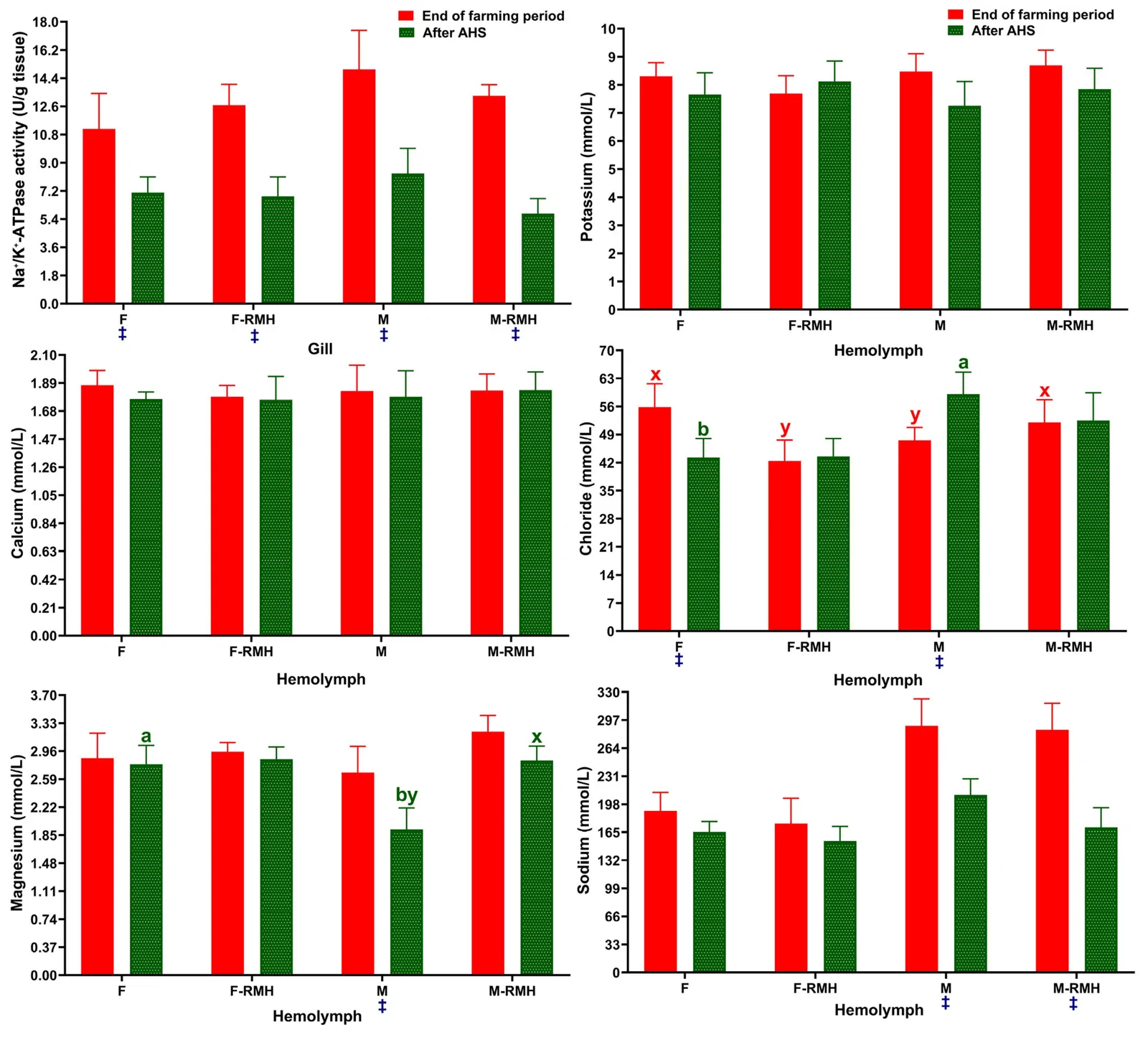
The results of ion homeostasis in green mud crabs reared under mild handling stress (RMH) and then exposed to acute handling stress (AHS). Values (mean ± SD) are from three samples per treatment, from three tanks. Each sample represents a tank, which was made by pooling two crab samples from each tank. Statistical analysis was performed at the level of the tank. Means without letter labels are not significantly different based on two-way ANOVA, and when the interaction was significant, the data were unpacked in the figures. The letters a and b indicate a significant difference between female (F) vs. male (M) or female-RMH (F-RMH) vs. male-RMH (M-RMH). The letters x and y indicate a significant difference between F vs. F-RMH or M vs. M-RMH. The symbol (‡) indicates significant differences between each group before and after AHS based on an independent-samples *t*-test (*p* < 0.05).

The data related to ion parameters, including Na^+^/K^+^-ATPase, sodium, potassium, calcium, magnesium, and chloride, were reported in Figure 8. After the rearing period, males had a higher level of sodium than females (*p* < 0.05). The interaction effect for chloride was significant. After AHS, males had higher values of sodium and chloride than females, and the Control group showed a decreased sodium level compared to the RMH treatment (*p* < 0.05). AHS also significantly decreased Na^+^/K^+^-ATPase levels in all groups. AHS also increased chloride and decreased magnesium and sodium in the M group (*p* < 0.05).

### 3.9. Gene Expression Related to Endoplasmic Reticulum Stress

**Figure 9:**
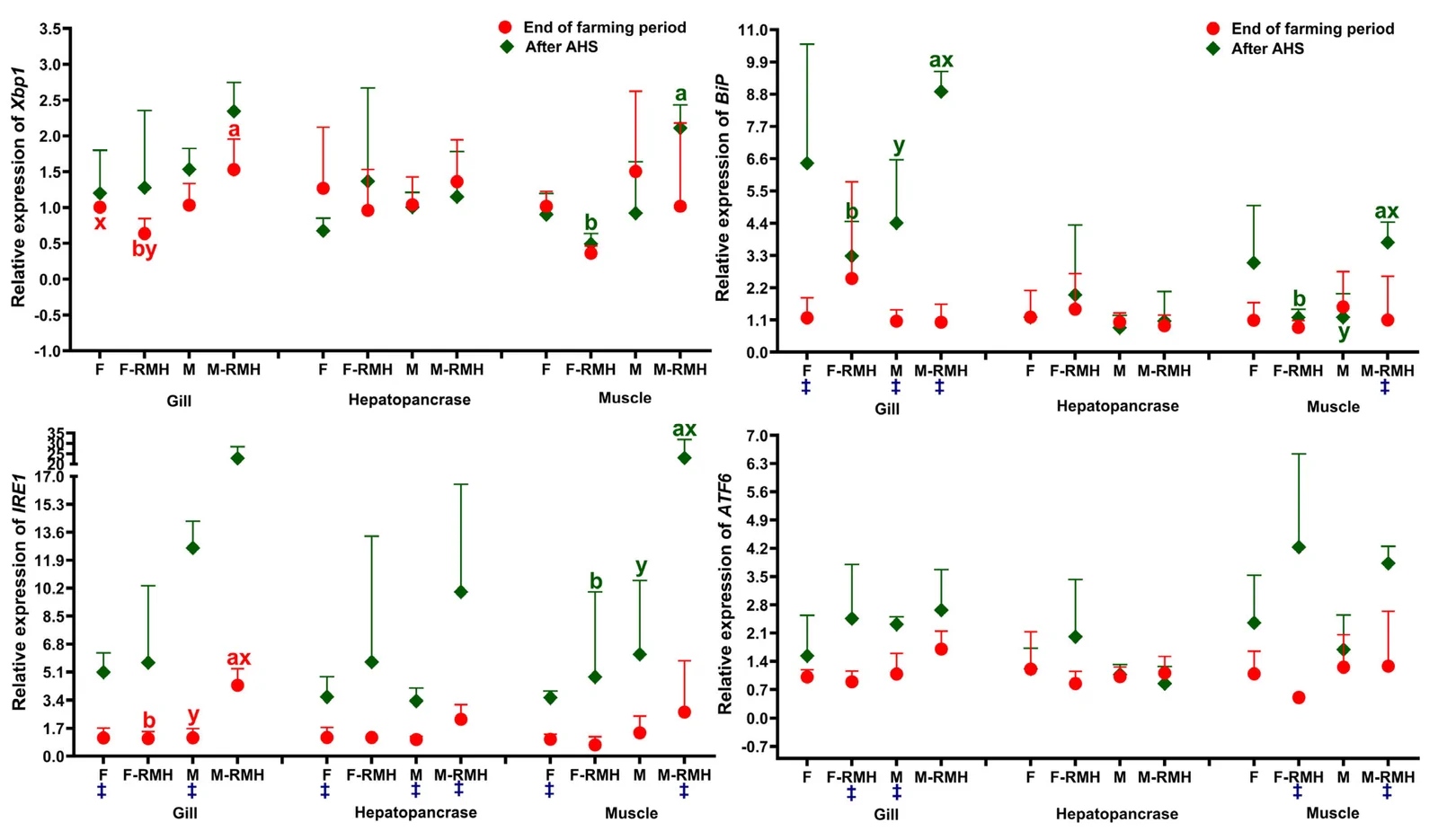
The results of gene expression related to endoplasmic reticulum stress in green mud crabs reared under mild handling stress (RMH) and then exposed to acute handling stress (AHS). Values (mean ± SD) are from three samples per treatment, from three tanks. Each sample represents a tank, which was made by pooling two crab samples from each tank. Statistical analysis was performed at the level of the tank. Means without letter labels are not significantly different based on two-way ANOVA, and when the interaction was significant, the data were unpacked in the figures. The letters a and b indicate a significant difference between female (F) vs. male (M) or female-RMH (F-RMH) vs. male-RMH (M-RMH). The letters x and y indicate a significant difference between F vs. F-RMH or M vs. M-RMH. The symbol (‡) indicates significant differences between each group before and after AHS based on an independent-samples *t*-test (*p* < 0.05).

**Figure 10:**
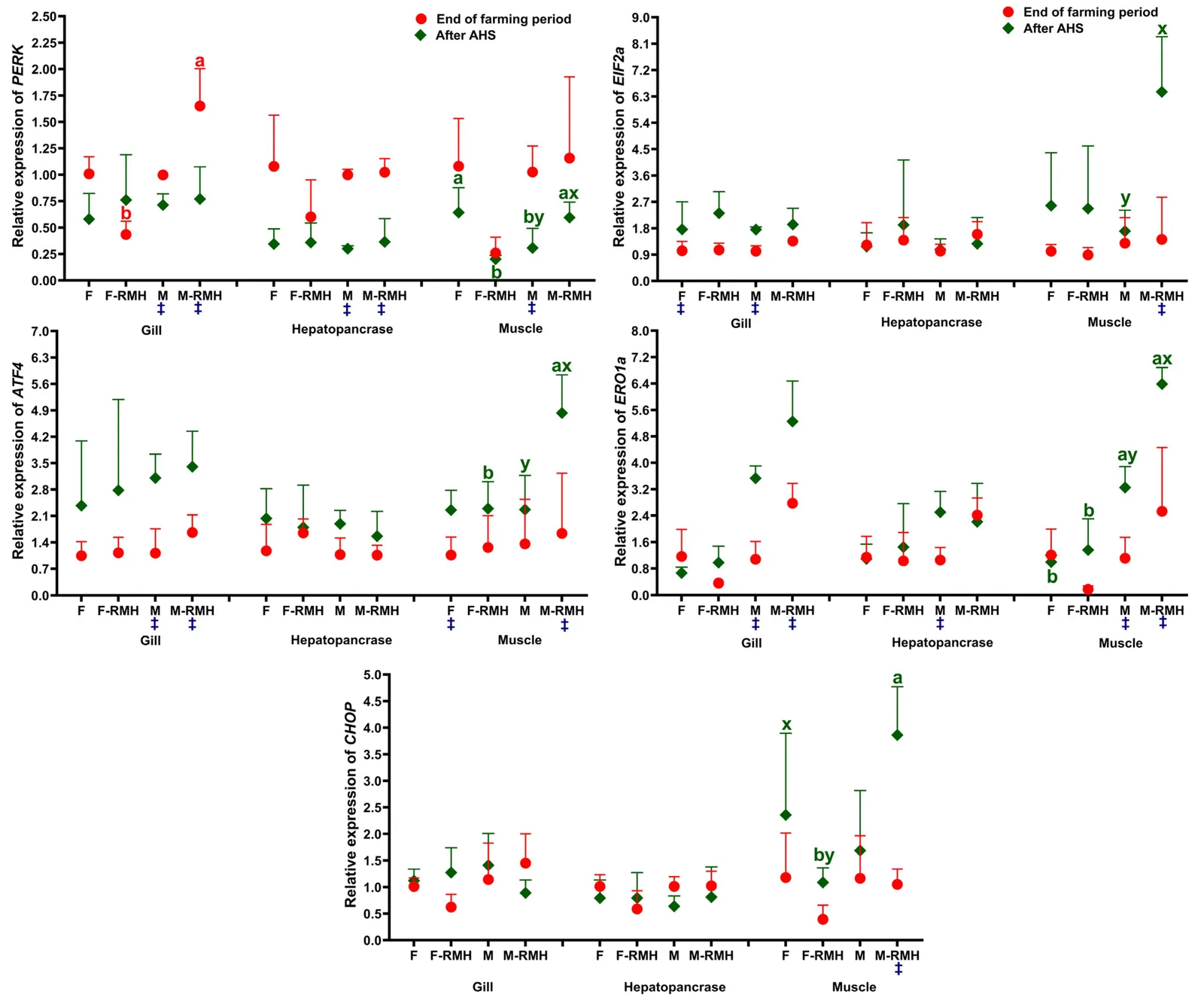
The results of gene expression related to endoplasmic reticulum stress in green mud crabs reared under mild handling stress (RMH) and then exposed to acute handling stress (AHS). Values (mean ± SD) are from three samples per treatment, from three tanks. Each sample represents a tank, which was made by pooling two crab samples from each tank. Statistical analysis was performed at the level of the tank. Means without letter labels are not significantly different based on two-way ANOVA, and when the interaction was significant, the data were unpacked in the figures. The letters a and b indicate a significant difference between female (F) vs. male (M) or female-RMH (F-RMH) vs. male-RMH (M-RMH). The letters x and y indicate a significant difference between F vs. F-RMH or M vs. M-RMH. The symbol (‡) indicates significant differences between each group before and after AHS based on an independent-samples *t*-test (*p* < 0.05).

The results of gene expression related to ER stress and the output of two-way ANOVA were reported in Table S1, Figure 9, and Figure 10. The results after 60 days of rearing indicated that the “sex effect” and “RMH effect” were not significant for any of the nine genes. The interaction effects for *XBP1*, *IRE1*, *ERO1α*, and *PERK* in the gill were significant (*p* < 0.05), as shown in Figure 9 and Figure 10.

After AHS, *IRE1* and *ERO1α* in the gill followed the same trend, and the RMH group had higher expression of these genes than the Control (*p* < 0.05). Further, males had significantly higher expression of these genes compared to females. The *ATF6* expression in the muscles of RMH groups was greater than that of Controls. “Sex effect” and/or “RMH effect” for other genes were not significant after AHS.

The clearest observation before and after AHS was related to upregulation of *XBP1*, *BIP*, *IRE1*, *ATF6*, *ATF4*, *ERO1a*, and *EIF2a*, especially in the gill and muscle of M and M-RMH groups. The M-RMH group for two of these genes had significantly higher expression after AHS compared to before AHS. However, in *PERK*, the opposite trend was observed, and crabs showed downregulation of this gene after AHS (*p* < 0.05).

## 4. Discussion

The current research indicated that while growth and survival rate were not affected by RMH after 60 days, the RMH group could have fewer broken legs and less aggressiveness after AHS. Further, females showed a higher survival rate than males after AHS (54.17% vs. 20.83%). The growth and behavior of this trial were already discussed [25]. Further, we could not find any clear relationship between transcriptomics [25] and classic parameters of the current study. The clearest change in the current data was related to energy shift and antioxidant parameters in the RMH group. The male M had lower values of total protein in the gill and higher levels of catalase and GST in muscle. Further, decreased lysozyme, peroxidase, and T-AOC levels and elevated total amino acids, LDH, sodium, and cholesterol in the M group were observed. The AHS changed the parameters in male groups more than in females, and perhaps this was the reason for the lower survival rate after AHS. In general, less responsiveness and physiological alteration in crabs, like other animals, when they encounter stress, can guarantee their survival rate and fitness. It should be noted that a higher sample size can strengthen the validity of results, and careful interpretation is required. The analyses are intended to remain exploratory and no multiplicity adjustment was applied, meaning that the reported *p* values are nominal, and that individual significant findings should therefore be interpreted cautiously because of the increased risk of false-positive results.

### 4.1. Stress Response

Stress response in crustaceans contains 3 main steps. The initial response is the rapid release of CHH from the sinus gland to support energy demand. The secondary response is characterized by elevated hemolymph glucose (via glycogen reserves), increased lactate, and other physiological and hemolymph changes, which usually come from metabolic acidosis and the accumulation of metabolic end products such as ammonia. The tertiary response is an alteration in energy homeostasis at the level of the whole animal, which affects feed intake, growth, the immune system, and disease resistance [3,4,28]. Elevated CHH and glucose are adaptive physiological responses that facilitate the restoration of homeostasis, but other physiological alterations, such as increased lactate and ammonia, are maladaptive [29]. In the current study, we tried to measure most of the parameters related to the stress response to understand the mechanisms behind the habituation. The clear shift in males under AHS was a depletion of glucose. This shows that crabs in this group tried to handle the stressful situation, but it seems that they were not able to do so, and energy depletion occurred. On the other hand, females were able to handle this situation by keeping homeostasis as unchanged as possible. The CHH and lactate did not change among the groups, which was surprising. CHH is sensitive to a wide range of stressors and increases with handling and trawling stress in Norway lobsters [30,31]. However, declining levels of this hormone in American lobster (*Homarus americanus*) exposed to shipping stress and high temperature [32] and no change [33] were also observed, showing that perhaps CHH is not directly related to stress and that factors such as the time of sampling, species, kind of stressors, and rapid degradation affect this hormone/neurotransmitter level. CHH is responsible for the regulation of glucose concentration in the hemolymph, and glucose and lactate control CHH release by negative and positive feedback, respectively [32,34,35,36]. Many stressful situations, such as frequent handling stress, result in hyperglycemia [16,37,38], and there is strong evidence that the production of CHH is the cause of this hyperglycemia [39,40]. The glucose level in the current research was depleted, and crabs could not activate the CHH channel. Another hypothesis is that, due to a lack of change in lactate and anaerobic metabolism, CHH also remained unchanged. This is unlike other studies in crustaceans, where chasing stress elevated lactate and glucose and also decreased triglycerides to provide energy for sustaining aerobic metabolism [7]. The third hypothesis is that perhaps we could not catch the CHH change, and 3 h were too late to monitor the CHH alteration, and crabs after 3 h of AHS were in recovery time. When the Christmas Island red crab (*Gecarcoidea natalis*) [41] and Christmas Island blue crab (*Discoplax celeste*) [42] were chased for 10 min, the elevation of circulating lactate coincided with an increase in the levels of CHH in the hemolymph. CHH, glucose, and lactate peaks under stressful conditions in other crustaceans seem to be less than 2 h [7,43,44]. However, the peak of these 3 parameters in crab studies with handling stress was 3–4 h [5], which was used as a reference for the current study. On the other hand, breakdown of glycogen occurred in the muscles of the Control male group but without a change in CHH, which is unlike other studies (see review [40,45]). Conclusively, the stress response and the role of the main drivers are complicated in crabs, and many more studies are required to gain insight into green mud crab stress physiology.

The increased LDH in the current study was in line with research in giant river prawn (*Macrobrachium rosenbergii*) after 30 min of handling stress [46]. Ammonia concentration in males in the current study was elevated, which shows a high breakdown of nitrogen and carbon compounds, such as amino acids, possibly for energy. Physical manipulation, such as capture, handling, and exercise, has been shown to increase hemolymph ammonia concentration for 1–3 h after stressors [3,47,48], which is in line with our data. While less focused in the literature, LDH and ammonia can be reliable biomarkers of stress in crabs.

### 4.2. Energy Homeostasis

In crustaceans, in order to provide energy, lipids and proteins can be metabolized by the aerobic pathways. Carbohydrates can be metabolized by aerobic and anaerobic routes [49,50]. The present study showed that all 3 nutrients were involved in providing energy for crabs after AHS in the male (M) group. As the survival rate of this group was the lowest, we can hypothesize that while crabs tried to use all available resources, they were not able to handle AHS, and possibly energy depletion occurred. Other studies also showed that various stressors can induce protein and lipid usage via gluconeogenesis to satisfy energy demand [37,38,51], as observed in our data. Unlike the current investigation, other researchers reported that lipid and protein were not used for energy during stress in crustaceans [43,44]. Not completely unexpectedly, we found that triglycerides were depleted after AHS in all 4 groups, which was in accordance with other studies in crustaceans [7,37,38]. Decreased total amino acids in the M group also suggests usage of protein to satisfy increased metabolic demand, which was similarly observed earlier in crustaceans [38,44]. Despite all changes, the crabs in the M treatment could provide enough ATP, as the ATP level in muscle did not differ among groups. After AHS, all groups could handle the situation without depleting the glycogen reserve, except the male Control group. Matching this data with the drop in amino acids and triglyceride levels shows more evidence of extreme stress levels in the male group. Aggressive behavior is energetically costly, and when we match this result with aggressiveness in males, energy depletion makes more sense.

### 4.3. Ion Homeostasis

Crustacean hemolymph contains inorganic ions, gases, digestive products, metabolic substrates, and waste products, and it is a physiologically significant tissue, especially for osmoregulatory purposes. Osmoregulatory capacity has been considered an indicator of stress response in crabs [52,53,54,55]. Na^+^/K^+^-ATPase is a crucial membrane-bound enzyme that is vital in preserving ionic and osmotic equilibrium in aquatic organisms. This enzyme actively extrudes sodium from the cell and imports potassium into the cell to sustain homeostasis and physiological stability [56]. Reduced Na^+^/K^+^-ATPase and sodium compromises osmoregulatory ability [57], and the decline in this enzyme by AHS in all 4 treatments shows this stress has been severe enough to disturb osmoregulation. Loss of ability to regulate hemolymph ions was reported in commercial shipments and hypoxia in lobsters and crabs [58,59], which is in line with the current study. Osmoregulation changes during stress can show the physiological status of crustaceans, and applying this technique more is recommended.

### 4.4. Health Status

Stress plays a very important role in crabs’ health and vulnerability to disease because it dysregulates their immune system response [3,4]. One of the clearest changes in immunity in the current study was increased ACP, lysozyme, and peroxidase in male groups by AHS. Further, after the farming period, males had lower lysozyme and peroxidase. This shows that chronic stress decreased these parameters, while short AHS increased lysozyme and peroxidase. Similar to our research, chronic stress inhibited immune responses due to an allostatic load and a deficit of the resources needed to support the immune system [60,61,62]. Similarly, increased peroxidase activity and lysozyme after 12 h of handling stress were observed. In this study, however, extending the stress period to 3 days impaired physiology and immunity [63]. Short-term acute handling stress stimulated lysozyme activity in rainbow trout (*Oncorhynchus mykiss*) [64], and plasma nitric oxide and peroxidase levels were elevated in the group most frequently subjected to stress [65]. However, in a few studies conducted in crustaceans, immune parameters such as total protein were not altered by long-term handling stress of whiteleg shrimp (*Penaeus vannamei*) [38,43]. While more studies are required in crustaceans, increased immunity, in theory, can be beneficial; it can also be physiologically demanding. Further, the stressor type and timeline play key roles in crabs’ response to stress and in inducing habituation; monitoring of immune system parameters is essential.

Acute stress can trigger oxidative stress and stimulate mitochondrial respiration, oxidative phosphorylation, and reactive oxygen species production, and reduce the antioxidant capacity [66]. In the present research, the clearest change due to AHS was observed in the M group, with a reduction in SOD and MDA levels in the 4 tissues, and GPx, T-AOC, and GST in some tissues. These results showed that the male group was the most sensitive to AHS, and due to the aggressiveness of this group (which perhaps causes oxidative stress), wasted energy to return homeostasis to the normal level may have occurred. This perhaps eventually led to an extreme reduction in the survival rate of M crabs. Similarly, increased GPx activity and SOD in the muscle of exhausted whiteleg shrimp [66] and in hypoxia–reoxygenation conditions [67] were observed. On the other hand, increased or no change in antioxidant defense parameters in other treatments of the current study shows that the antioxidant response apparently sufficed to prevent oxidative damage in the tissue of the green mud crabs. This phenomenon was earlier observed in the escape response or recovery in whiteleg shrimp [68]. Researchers further indicated that increased activity of GPx and SOD, with no change in catalase, protein carbonyl, and reactive oxygen species production 1 h after exhaustion, which, for some parameters, is similar to the present data. No change in superoxide anion production and SOD activity with repeated handling stress in whiteleg shrimp was reported [38]. In general, a lack of change in antioxidant parameters shows that animals were able to handle stressful situations and keep reactive oxygen homeostasis balanced, as observed in most treatments in the current study but not the M group. Further, it seems that the energy budget in these groups (except M) might be sufficient to accommodate the increased energy demands of an immune and antioxidant elevation caused by AHS.

Regarding sex, the present study clearly showed a distinct response of the immune system and antioxidant defense between males and females, which perhaps comes from the extreme aggressiveness of males and the reproduction process of females. Similarly, males of the freshwater prawn (*Macrobrachium potiuna*) had lower expression of antioxidant genes, but females had higher levels of those genes under a stressful condition [69]. Males of *Callinectes amnicola* and *Geothelphusa dehaani* showed more serious oxidative damage and, therefore, their population was reduced over 24 months [70]. A distinct response to oxidative stress, especially lipid peroxidation (ferrous oxidation-xylenol orange method) in the hepatopancreas, gill, and hemolymph of male and female crabs (*Platyxanthus orbignyi*) was observed due to a short 4 h air exposure [71]. Similarly, the calanoid copepod (*Pseudodiaptomus annandalei*) male was almost twice as sensitive as the female, measured by the median lethal concentration (LC50) of cadmium after 96 h [72]. Considering the sex impact in any experimental design that affects the immune system and antioxidant defense in crabs is recommended.

### 4.5. Expression of Genes Associated with ER Stress

ER stress is one of the most prevalent and regular types of internal stress at the cellular level, and many studies in aquaculture have shown that ER stress can be manifested by a variety of external stressors [73]. The most common approach for monitoring ER stress has been testing relative gene and protein expression. In terms of cell signaling for ER stress, the most common gene indicators identified in aquaculture species include *BIP*, *IRE1*, *PERK*, *ERO1a*, *ATF4*, *ATF6*, *XBP1*, and EIF2a [73]. The results of the current investigation showed that at the end of the 60-day trial, and after AHS, no genes from the hepatopancreas were changed, and only *IRE1* and ERO1a in the gill and *ATF6* in muscle were significantly higher in RMH groups. Male green mud crabs also showed higher expression of these genes than females. Comparing the data before and after AHS showed that male RMH had upregulated expression for most genes (but not *PERK*). It seems that males of this group used this upregulation to tackle AHS. Females, however, seemed to experience less stress, and these genes did not need to be upregulated. Male crabs are very aggressive, and it is not surprising to see that ER stress was induced in the present research. The survival rate of females after 8 days of AHS was higher than that of males, and this is further evidence of the different stress responses of females. As far as we know, no investigation has tested ER stress and related pathways under handling stress in aquatic species, let alone the impact of sex on this process. Considering other stressors, expression of the *IRE1*, *ATF6*, and *XBP1* genes was elevated after 24 h of ammonia stress in the swimming crab (*Portunus trituberculatus*) [74]. Many more studies are required to test the effect of sex on the stress physiology and ER stress of crustaceans, considering the aggressiveness of crab males and female reproduction and molting processes. ER stress can crosstalk with energy homeostasis, stress responsiveness, and oxidative stress, indicating that the investigation of these mechanisms simultaneously is required to understand various phenotypes.

## 5. Conclusions

The current investigation demonstrated that RMH did not affect females; males were less aggressive, and the M-RMH group had a higher survival rate after AHS (33.0%) compared to males (M group 9.09%). The behavior of the M-RMH group can be cautiously interpreted as habituation occurrence, and physiological and survival outcomes were associated responses. Consequently, the low survival rate after AHS and the lack of habituation in the M group coincided with lower values of total protein in the gill; lysozyme, peroxidase, T-AOC, and glucose in the hemolymph; and higher levels of LDH, sodium, and cholesterol in the hemolymph. As male crabs have been more vulnerable to handling stress than females, considering the sex effect in stress studies is suggested. A higher sample size is recommended for increasing statistical power. Experimental design affects the outputs considerably and thus, testing more diverse experimental designs is suggested. More research is required on the use of “stress programming” to habituate crustaceans and lessen their aggression. As aggressiveness has been one of the main obstacles in crab aquaculture, habituation can pave the way toward optimizing rearing conditions.

## Figures and Tables

**Table 1:** The genes, their sequences (5′-3′), and corresponding sizes (bp).

Gene	Sequences (5′-3′)	Sizes
*ATF6* F	CTGAGGCCCTGCAAGTTGAT	206 bp
*ATF6* R	GTTGGCATAAGAAGTGACATACGAG	
*IRE1* F	CACCGTGACATTAAGCCTCATA	368 bp
*IRE1* R	ATCCTTTCAATCAGGGTAGCAG	
*EIF2α* F	CCAGAGAGGAGCAAGGAGT	158 bp
*EIF2α* R	GCAGCAGAGAAAGGAACAA	
*XBP1* F	CCTGTGTCACCTCTCTTGGGGG	386 bp
*XBP1* R	TCCTTGCTGCTGGTCATGCTCT	
*PERK* F	ACGCAGCCCACTATCAGC	171 bp
*PERK* R	GCCATCAATCACTCCCAC	
*ERO1α* F	CGGAAGGCAGTGAGTATGTGG	266 bp
*ERO1α* R	GAGGAGGTAGCGAGCAGACAA	
*BIP* F	AGCGGCTCATCGGTAGGGAA	252 bp
*BIP* R	CGTTGAAGTAGGCAGGGACA	
*ATF4* F	TCCTCTTGTCCCAGTTCCC	374 bp
*ATF4* R	TGCTGTTGCTGCTTATCATCT	
*CHOP* F	GTCCATCACGGAAGAAGAGG	133 bp
*CHOP* R	AGAGTAGGGCGAGGCAAC	
18S rRNA F	ATGATAGGGATTGGGGTTTGC	99 bp
18S rRNA R	AGTAGCGACGGGCGGTGT	

## Data Availability

The data presented in this study are available on request from the corresponding author due to privacy.

## Supplementary Materials

The following supporting information can be downloaded at: https://www.mdpi.com/article/10.3390/ani16193143/s1, Figure S1: Supplementary file 1: experimental design; Table S1: The results of two-way ANOVA analysis with SPSS for measured parameters. When the interaction was not significant, we compared the “Repeated Mild Handling Stress (RMH) Effect” and the “Sex Effect” via an independent-samples *t*-test at the end of the experiment and after the final acute handling stress (AHS) for the case these effects were significant (*p* < 0.05). Non-significant parameters were not reported.

## Abbreviations

RMH: repeated mild handling; AHS: acute handling stress; F: female; M: male; F-RMH: female-repeated mild handling; M-RMH: male-repeated mild handling; DNA: deoxyribonucleic acid; RNA: total ribonucleic acid; ATP: adenosine triphosphate; ACP: acid phosphatase; NOS: nitric oxide synthase; SOD: superoxide dismutase; MDA: malondialdehyde; GPx: glutathione peroxidases; GST: glutathione transferases; ALP: alkaline phosphatase; AST: aspartate aminotransferase; ALT: alanine aminotransferase; LDH: lactate dehydrogenase; CHH: Crustacean hyperglycemic hormone; ER: endoplasmic reticulum; *CHOP*: C/EBP homologous protein; *ATF6*: activating transcription factor 6; *IRE1*: inositol-requiring protein-1; *EIF2α*: eukaryotic translation initiation factor 2-alpha; *XBP1*: X-box binding protein 1; *PERK*: protein kinase RNA-like ER kinase; *ERO1α*: endoplasmic reticulum oxidoreductase-1; *BIP*: immunoglobulin heavy chain binding protein; *ATF4*: activating transcription factor 4.
