# Supplementary material for: Male Green Mud Crab (Scylla paramamosain) Is More Vulnerable to Handling Stress than Female: A Comprehensive Insight into Physiological Changes and Health Status
Source: Animals (Basel). 2026 Oct 8;16(19):3143. doi: 10.3390/ani16193143 (PMC13663459; doi:10.3390/ani16193143)
Supplement: Supplementary file 1 [file animals-16-03143-s001.zip › animals-4533340-supplementary.pdf]

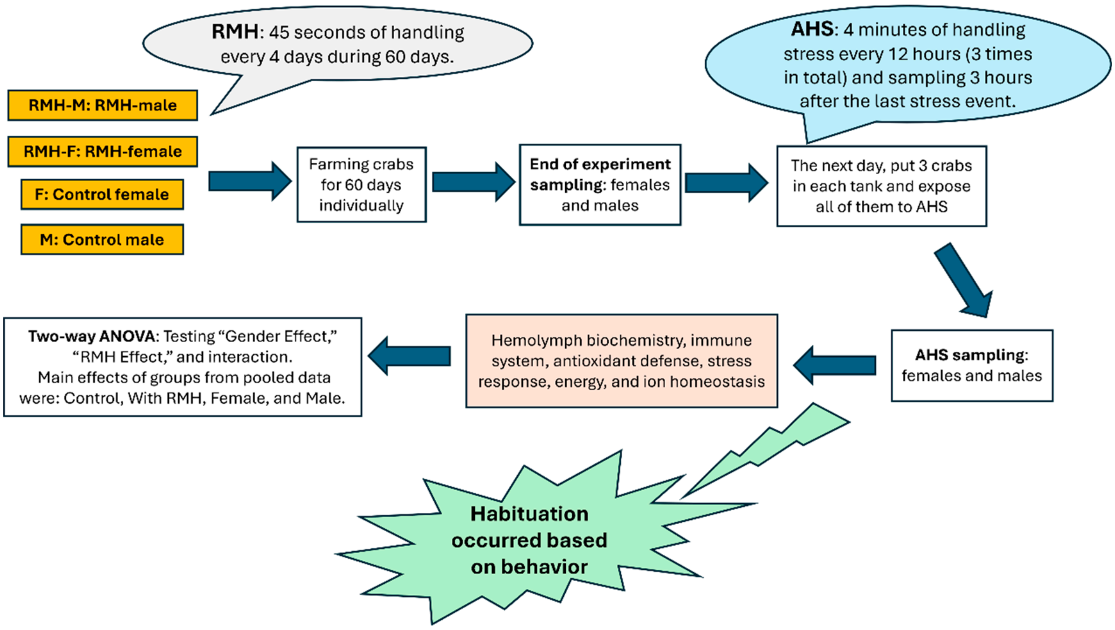

Figure S1: Supplementary file 1: experimental design

Table S1. The results of two-way ANOVA analysis with SPSS for measured parameters. When the interaction was not significant, we compared the "Repeated Mild Handling Stress (RMH) Effect" and "Sex Effect" via Independent Samples T-Test for the end of the experiment and after final acute handling stress (AHS) for the case these effects were significant (P<0.05). The non-significant parameters were not reported.

		P-value			Main effects (mean)			
		**RMH Effect**	**Sex Effect**	**Interactions**	**Control**	**With RMH**	**Female**	**Male**
End of experiment (60 days farming)	Survival rate after 8 days AHS	0.563	**0.042**	0.262			54.17	20.83
	Total protein-gill	0.137	0.01	0.426			1.12	0.86
	Ammonia	0.001	0.881	0.962	27.82	18.00		
	BUN	0.001	0.105	0.594	9.26	5.70		
	Lysozyme	0.869	0.002	0.152			894.95	587.74
	NOS	0.144	0.027	0.891			5.18	6.69
	Peroxidase	0.027	0.001	0.786	10.44	8.32	11.88	6.88
	CAT-hepatopancreas	0.002	0.098	0.594	25.09	33.28		
	CAT-muscle	0.247	0.037	0.998			11.63	14.08
	MDA-serum	0.756	0.045	0.876			430.10	324.37
	MDA-hepatopancreas	0.023	0.013	0.689	417.42	512.19	518.13	411.48
	GPx-hepatopancreas	0.033	0.44	0.051	1052.9	874.35		
	GST-muscle	0.133	0.005	0.972			28.23	40.43
	Total antioxidant-serum	0.213	0.024	0.051			95.09	78.75
	Lactate muscle	0.022	0.409	0.409	5.60	6.38		
	Triglyceride	0.003	0.809	0.153	9.77	15.85		
	Glycogen	0.001	0.160	0.160	3.93	2.51		
	ATP	0.047	0.845	0.087	515.59	403.17		
	Total amino acid-serum	0.001	0.001	0.059	8.19	4.82	4.71	8.31
	LDH	0.001	0.012	0.157	44.61	27.15	31.60	40.15
	Sodium	0.580	0.001	0.769			182.66	287.86
After	Total protein-gill	0.017	0.129	0.114	0.76	0.58		
	Ammonia	0.008	0.07	0.296	37.47	28.21		

ACP	0.002	0.001	0.094	0.32	0.48	0.32	0.49
CAT-muscle	0.299	0.002	0.057			11.63	16.52
MDA-gill	0.390	0.005	0.197			270.87	162.87
MDA-hepatopancreas	0.005	0.009	0.267	282.80	390.54	384.28	289.06
GST-muscle	0.001	0.273	0.120	17.98	42.04		
Total antioxidant-hepatopancreas	0.048	0.770	0.072	221.12	282.81		
Total antioxidant-serum	0.036	0.001	0.731	77.35	93.68	104.02	67.01
Total antioxidant-gill	0.001	0.043	0.073	395.19	551.23		
Glucose	0.048	0.001	0.469	1.26	1.71	2.14	0.84
Cholesterol	0.075	0.001	0.097			0.62	4.67
Triglyceride	0.004	0.252	0.803	3.95	5.95		
ATP	0.013	0.578	0.152	784.70	576.43		
Chloride	0.348	0.004	0.310			43.39	55.80
Sodium	0.048	0.022	0.235	187.26	162.67	160.03	189.90
IRE1-gill	**0.039**	**0.001**	0.058	8.87	14.24	5.37	17.73
ERO1a-gill	**0.034**	**0.001**	0.117	2.09	3.11	0.82	4.39
ATF6-muscle	**0.036**	0.524	0.872	2.02	4.03		

The interaction effects for protein carbonyl, ALP, SOD, total antioxidant-hepatopancreas, total antioxidant-gill, total antioxidant-muscle, GST-hepatopancreas, glucose, cholesterol, Cl, XBP1-gill, IRE1-gill, ERO1a-gill, and PERK-gill after 60 days rearing were significant which unpacked in figures. Regarding after AHS, total protein-hepatopancreas, total protein-muscle, uric acid, blood urea nitrogen, lysozyme, NOS, POD, protein carbonyl, ALP, MDA-muscle, GPx-gill, SOD, CAT-hepatopancreas, MDA-serum, GPx-hepatopancreas, GPx-gill, GST-muscle, GST-hepatopancreas, total antioxidant-muscle, MDA-gill, total amino acid-serum, total amino acid-hepatopancreas, LDH, Mg, *XBP1*-muscle, *EIF2a*-muscle, *BIP*-gill, *BIP*-muscle, *IRE1*-muscle, *ATF4*-muscle, *CHOP4*-muscle, *ERO1a*-muscle, and *PERK*-muscle were significant which unpacked and shown in Figures.
